# Repair of the *TGFBI* gene in human corneal keratocytes derived from a granular corneal dystrophy patient via CRISPR/Cas9-induced homology-directed repair

**DOI:** 10.1038/s41598-017-16308-2

**Published:** 2017-12-01

**Authors:** Yukako Taketani, Kohdai Kitamoto, Toshihiro Sakisaka, Mikiko Kimakura, Tetsuya Toyono, Satoru Yamagami, Shiro Amano, Masahiko Kuroda, Tara Moore, Tomohiko Usui, Yasuo Ouchi

**Affiliations:** 1 Department of Ophthalmology, Graduate School of Medicine, University of Tokyo, Tokyo, Japan; 2 Nihon University Itabashi Hospital, Tokyo, Japan; 3 Inoue Eye Hospital, Tokyo, Japan; 4 Department of Molecular Pathology, Tokyo Medical University, Tokyo, Japan; 5 Biomedical Sciences Research Institute, Centre for Molecular Biosciences, Ulster University, Coleraine, Northern Ireland; 6 Avellino Labs, Menlo Park, CA USA; 7 Department of Mucosal Immunology, School of Medicine, Chiba University, Chiba, Japan

## Abstract

Granular corneal dystrophy (GCD) is an autosomal dominant hereditary disease in which multiple discrete and irregularly shaped granular opacities are deposited in the corneal stroma. GCD is caused by a point mutation in the *transforming growth factor-β-induced* (*TGFBI*) gene, located on chromosome 5q31. Here, we report the first successful application of CRISPR-Cas9-mediated genome editing for the correction of a *TGFBI* mutation in GCD patient-derived primary corneal keratocytes via homology-directed repair (HDR). To correct genetic defects in GCD patient cells, we designed a disease-specific guide RNA (gRNA) targeting the R124H mutation of *TGFBI*, which causes GCD type 2 (GCD2). An R124H mutation in primary human corneal keratocytes derived from a GCD2 patient was corrected by delivering a CRISPR plasmid expressing Cas9/gRNA and a single-stranded oligodeoxynucleotide HDR donor template *in vitro*. The gene correction efficiency was 20.6% in heterozygous cells and 41.3% in homozygous cells. No off-target effects were detected. These results reveal a new therapeutic strategy for GCD2; this method may also be applicable to other heredity corneal diseases.

## Introduction

Granular corneal dystrophy (GCD) is a bilateral, progressive, genetic, and non-inflammatory disease characterised by multiple granular deposits in the corneal stroma. Using the IC3D classification^1^, GCD has two subtypes, both of which are classified as Category 1, i.e., causal point mutations have been identified in the *transforming growth factor-beta-induced* (*TGFBI*) gene, located on chromosome 5q31^2^. TGFBI, also called keratoepithelin or Big-h3, is 68-kDa protein found in the extracellular matrix of human tissues. It is particularly abundant in the cornea.

There are two clinical types of GCD, GCD1 and GCD2. Although originally described in a family from the Italian region of Avellino, the R124H mutation associated with GCD2 is occurs in unrelated individuals in all populations studied and is the most common type in Asia, including Japan^3,4^. In GCD2, discrete grey-white granular deposits (hyaline) with snowflake, star, or disk shapes are detected in the corneal stroma at an early age^5^ and amyloid deposits are observed in elder patients in deeper stroma^6^. GCD2 has a diffuse anterior stromal haze between the typical granular opacity. The haze may be caused by amyloid deposits, which are thought to be similar to Gelatinous drop-like CD, instead of the linear opacity seen in the early stage of lattice dystrophy^6,7^. Compared to heterozygous patients, homozygous patients may have an onset under 10 years old, and demonstrate a more rapid progression. These progressive corneal opacities cause a loss of visual acuity. To avoid visual impairment in GCD2, phototherapeutic keratectomy (PTK) is a major treatment option. However, multiple opacities usually recur within several years^8^. Compared with PTK, corneal clarity can be retained for longer durations using keratoplasty, but opacity eventually occurs via the gradual invasion of host corneal cells, especially in homozygous patients^9^. Thus, the development of a radical treatment is needed. GCD2 is typically associated with an R124H (histidine replacing arginine) point mutation in the *TGFBI* gene; accordingly, a gene therapy approach may be effective.

CRISPR/Cas9 (clustered, regularly interspaced short palindromic repeats (CRISPR)/CRISPR-associated protein)-mediated genome editing has been increasingly applied to repair mutated genome sequences^10^. This versatile tool for genome engineering enables the induction of site-specific double-strand breaks (DSBs) using guide RNAs (gRNAs)^11–15^. DSBs can be repaired by two major pathways, non-homologous end joining and homology-directed repair (HDR). In the presence of exogenous donor DNA as a repair template, DSBs can be repaired precisely via the HDR pathway. This technique is useful for codon replacements or reporter insertions^16,17^. For small genetic modifications, such as point mutations, the application of single-stranded oligodeoxynucleotides (ssODNs) as HDR templates shows higher editing efficiency than that of plasmid donors^18^. Here, we report the first CRISPR-mediated HDR using cultured corneal keratocytes derived from an R124H GCD2 patient. The results of this study have important clinical implications given the lack of effective treatment options for GCD2.

## Results

### Gene targeting strategy and construction for CRISPR/Cas9-mediated HDR of an R124H mutation

**Figure 1:**
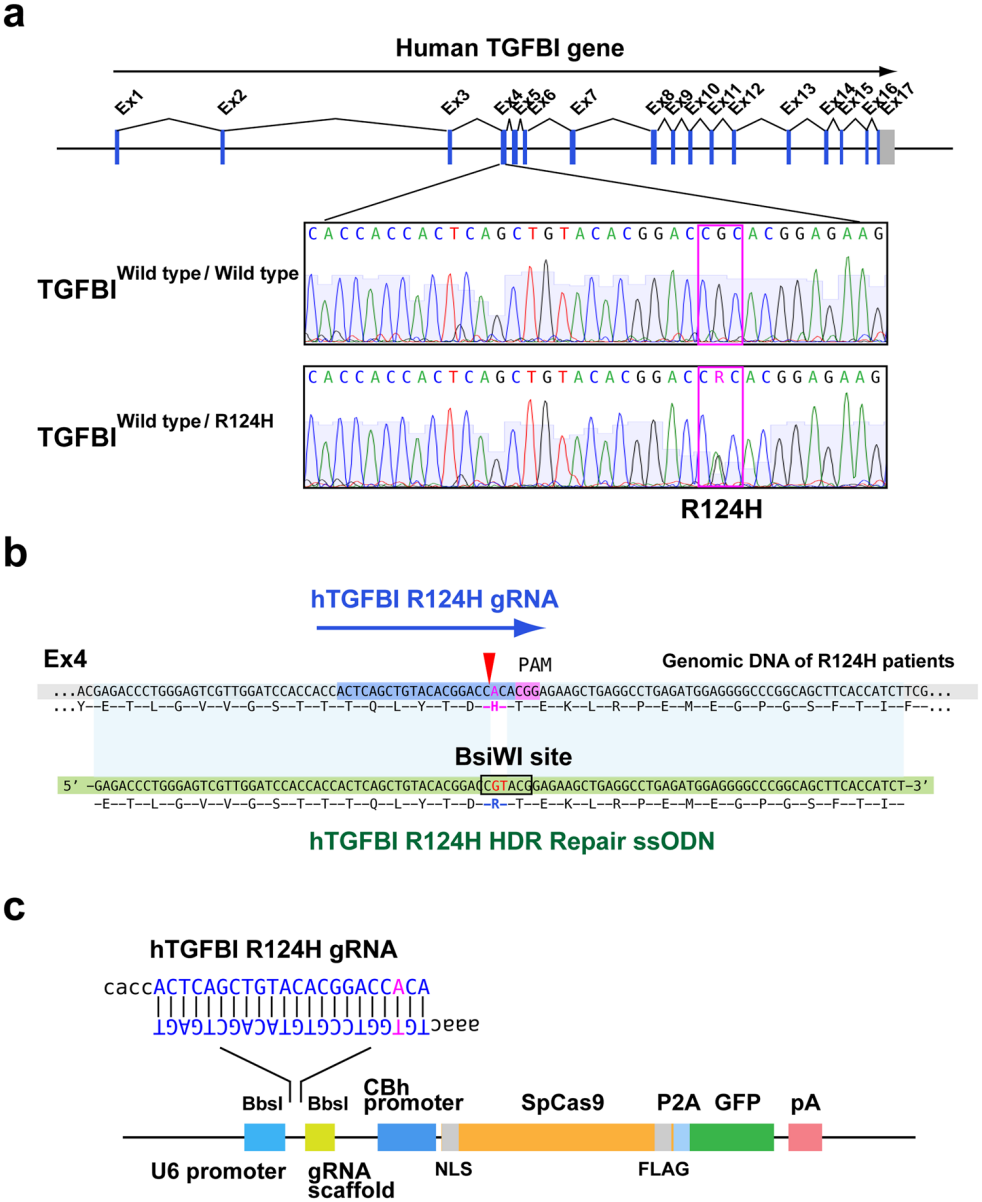
Gene targeting strategy for CRISPR/Cas9-mediated HDR of a TGFBI R124H mutation. **(a)** Schematic diagram of the *TGFBI* mutation in GCD2 in humans. (**b)** In GCD2, the 124^th^ protein position is histidine (H), instead of arginine (R). The recognition sight of donor single-strand DNA is also shown. **(c)** Linear structure of the plasmid transfected into R124H mutant cells. The plasmid (px 458) includes guide RNA targeting R124H mutant cells, Cas 9 protein sequences, and EGFP. TGFBI, transforming growth factor β-induced; GCD2, granular corneal dystrophy; HDR, homology-directed repair.

To develop an efficient strategy to repair the genetic mutation in GCD using CRISPR/Cas9, we used human cultured corneal keratocytes derived from an R124H GCD2 patient as a model system. The TGFBI R124H mutant keratocytes have a monoallelic point mutation at Arg124 (GCA→ACA) in Exon 4 of *TGFBI* (Fig. 1a). To repair mutant R124H cells, we designed an R124H mutation-specific gRNA based on a public algorithm (Fig. 1b). Then, the designed gRNAs were computationally evaluated for potential off-target effects using the E-CRISP algorithm. The gRNA with the lowest off-target risk was selected for subsequent analyses.

For the HDR repair template, we synthesized a 100-nucleotide (nt) donor repair template ssODN with a novel BsiWI restriction site (Fig. 1b). The substitutions ensured that the sequence of the wild-type donor template was resistant to CRISPR/Cas9 cleavage by the R124H mutation-specific gRNA, and the BsiWI restriction site allowed the tracking of HDR by restriction fragment length polymorphism (RFLP) (Fig. 1b). A pair of annealed oligos encoding a target sequence of R124H mutation-specific gRNA was cloned into the px458 vector, which enabled bicistronic expression of *Streptococcus pyogenes* Cas9 (spCas9) and green fluorescence protein (GFP) (Fig. 1c).

### CRISPR/Cas9-mediated HDR of an R124H mutation in human corneal keratinocytes

**Figure 2:**
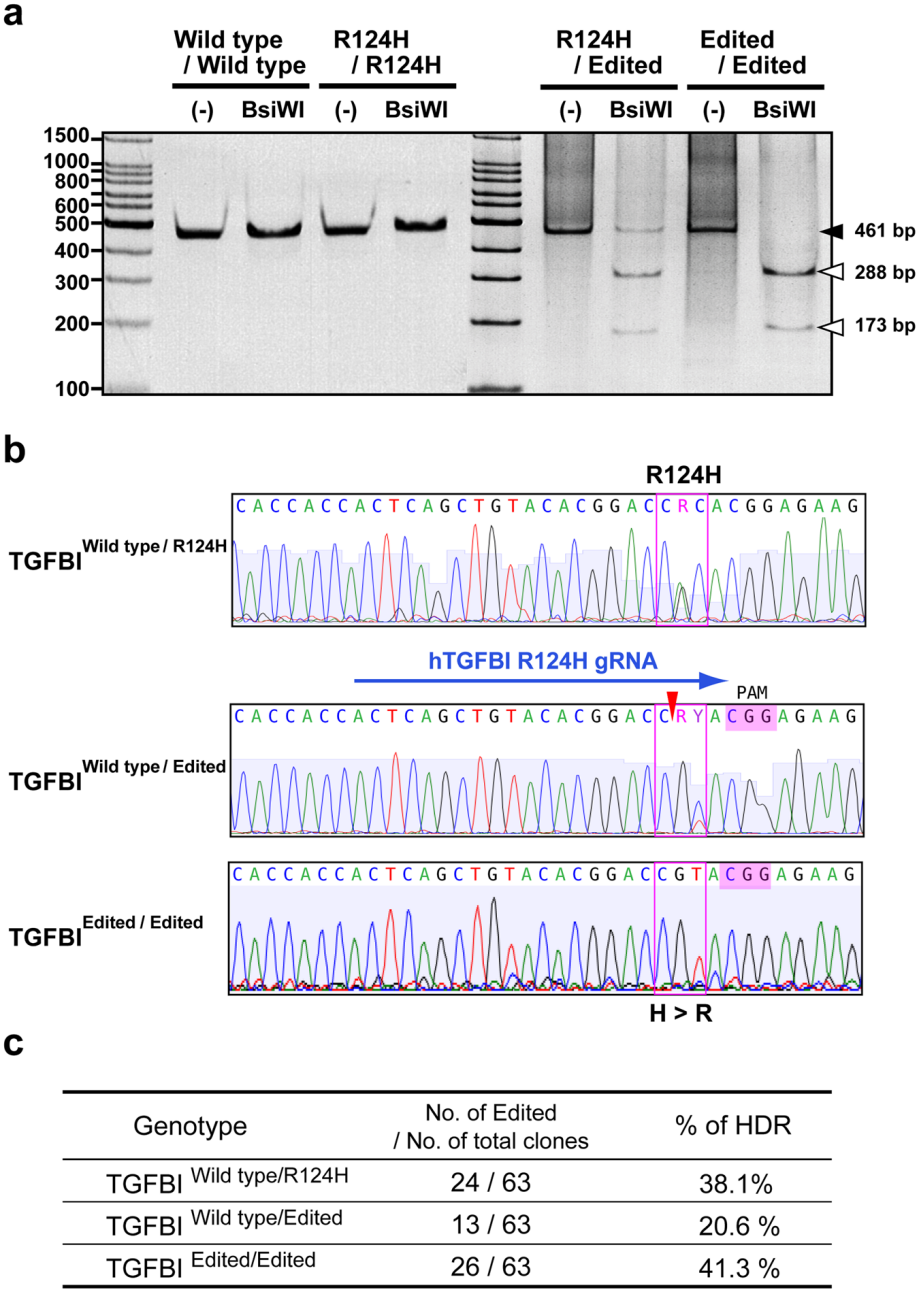
Correction of the mutation in TGFBI R124H mutant keratocytes using CRISPR-mediated HDR. **(a)** Result of an RFLP analysis of edited R124H cells. *TGFBI* exon 4 was amplified by PCR, and the products were treated with the BsiWI restriction enzyme. The lane with three bands was edited heterozygously and the lane with two bands was edited homozygously. **(b)** DNA sequences of PCR products amplified from the *TGFBI* gene of wild-type cells, a heterogeneous R124H mutant, and a repaired allele by HDR after transfection of Cas9 guide RNA and ssDNA. Two peaks were observed in the sequence of the R124H heterogeneous mutant, while the base of HDR-repaired cells was corrected to T. **(c)** Editing efficiency of CRISPR/Cas9-mediated HDR of an R124H mutation. RFLP, restriction fragment length polymorphism TGFBI, transforming growth factor β-induced; HDR, homology-directed repair.

The CRISPR plasmid expressing spCas9/gRNA was co-transfected into primary R124H mutant human corneal keratinocytes with the ssODN as a donor template. After 7 days, single GFP-expressing cells were harvested, added to individual wells of a 96-well plate, and clonally expanded. Then, the presence of a novel BsiWI restriction site was examined by RFLP-based genotyping. Genomic PCR products for wild-type alleles were not cleaved by BsiWI (Fig. 2a). However, genomic PCR products for several transfected colonies were cleaved by BsiWI, suggesting target site alterations by HDR (Fig. 2a). We also confirmed the genomic sequences of the PCR products (Fig. 2b).

The sequence of wild-type cells had CGC, specifying arginine, at the 124^th^ amino acid, and R124H mutant cells had CAC at this position. Neither is expected to be cleaved by BsiWI; however, gene-edited cells have CGT, which is expected to be cut at CGTAC. In an RFLP assay, we detected cells with heterozygous and homozygous editing, as shown in Fig. 2c.

### Efficiency of Cas9-mediated genome editing of the TGFBI R124H mutant gene

To examine the editing efficiency of the R124H mutant *TGFBI* gene, genomic DNA was extracted from the clonally expanded cells in 96-well plates and examined by RFLP-based genotyping. Owing to the low growth rate and viability of flow cytometry-sorted primary keratinocytes, not all cells were sufficiently expanded by single-cell cloning in 96-well plates. Cell growth and gene editing efficiency are summarised in Fig. 2c. Thirty-eight out of 192 clones were sufficiently expanded and examined by RFLP. Among all examined clones, 20.6% exhibited monoallelic *TGFBI* correction and 41.3% showed biallelic correction. Accordingly, 62% showed clear TGFBI R124H allele correction derived from the HDR template.

### Analysis of off-target cleavage by R124H mutation-specific gRNA

**Figure 3:**
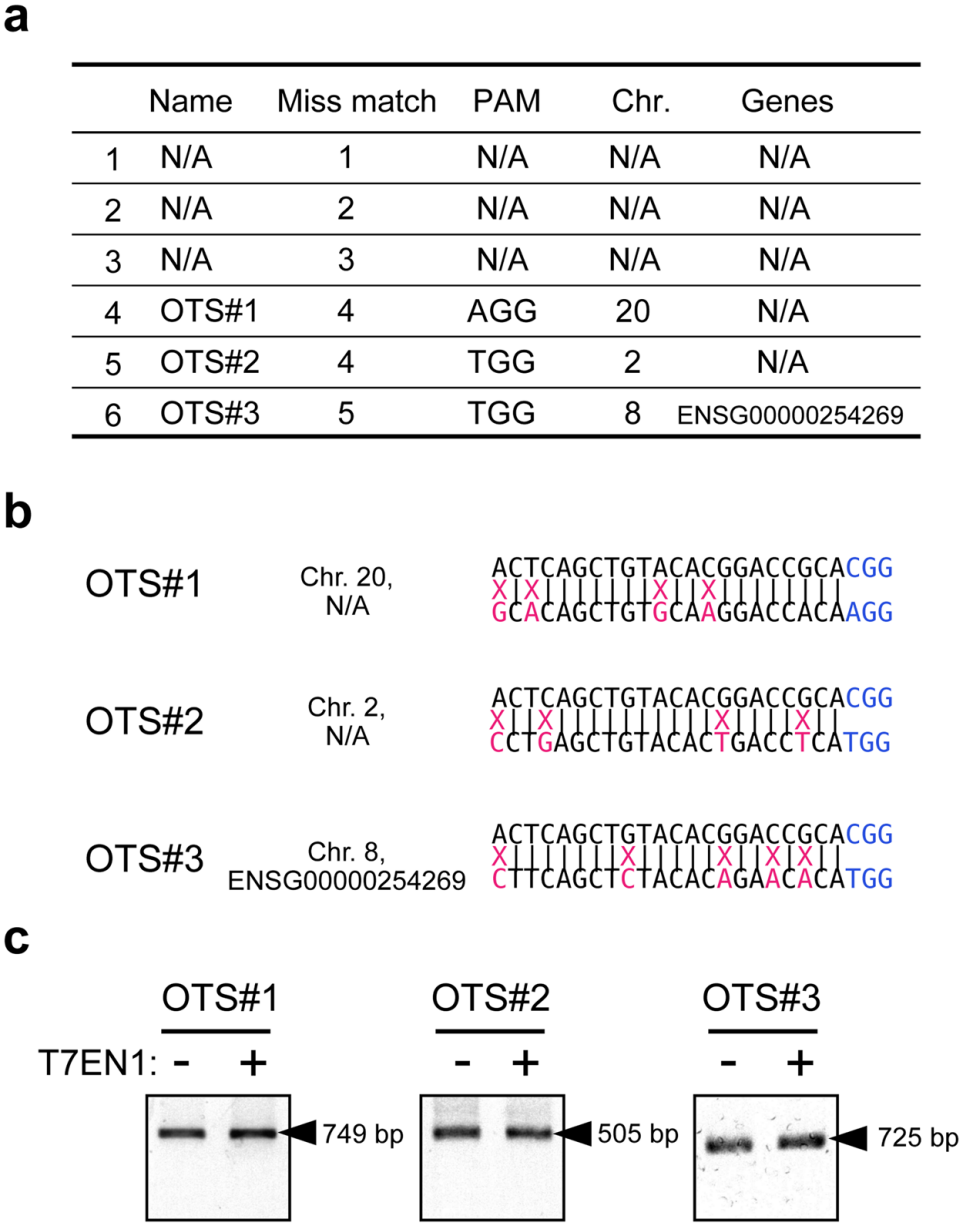
Off-target sites and T7 endonuclease cleavage assay of potential off-target loci. **(a)** Ranked list of potential off-target loci for R124H mutation-specific gRNA. Number of mismatched bases, PAM sequence, chromosomes, and target gene are indicated. **(b)** Sequence alignments of R124H mutation-specific gRNA and potential off-target loci. **(c)** Potential off-target sites in edited cells were amplified by PCR. After T7 endonuclease treatment, no off-targets effects were found at any site. N/A: not applicable.

To evaluate off-target effects mediated by the gRNA, a T7 endonuclease (T7EN1) cleavage assay was used to assess off-target cleavage. Since we rigorously designed and selected a *TGFBI*-specific gRNA to reduce the risk of off-target effects, only 3 potential off-target sites were found for the gRNA (Fig. 3a,b). We could not find any potential off-target sites (OTS) with mismatches of less than 3 nt. The 3 potential OTS had mismatches of more than 4 nt with the *TGFBI* gRNA (Fig. 3b). In the T7EN1 cleavage assay, we did not detect any off-target effects at the 3 OTS (Fig. 3c).

## Discussion

Current therapeutic modalities for GCD, i.e. PTK and keratoplasty, are invasive and are associated with frequent recurrence. The correction of *TGFBI* mutations in the local cornea may be a radical treatment for GCD patients, minimizing progression and the recurrence of corneal opacities. In this study, we successfully repaired point mutations in R124H mutant cells using CRISPR/Cas9 and HDR *in vitro*, without detectable off-target effects.

The CRISPR/Cas 9 system is an efficient tool for genome engineering and disease treatment. Kaminski *et al*. successfully eliminated HIV genomes in human T cells *ex vivo* ^19^; they reported a low editing efficiency in primary culture, even using a lentivirus delivery system^19^. Similarly, the low transfection and growth efficiency in this study (Fig. 2c) may be attributed to the use of primary culture cells. However, in general, plasmid transfection may be safer than viral transfection *in vivo*. Fortunately, despite the low growth rate, our results reveal that the efficiency of CRISPR/Cas9 in gene correction was higher compared with those of previous studies^15^. It is reported that the efficacy of HDR is generally not high, however, the efficiency of HDR using asymmetric donor DNA is much higher (maximum 60%) than that of conventional HDR^20^. In this study, the efficacy of HDR using ssODN was greater than 60%. The reason for the high efficiency of HDR in our study is unclear, but may be explained by the unique characteristics of the DNA repair ability of corneal epithelial cells. Previously, Mallet *et al*.^21^ demonstrated that DNA damage in human corneal epithelial cells by ultraviolet radiation could be repaired faster than that in epidermal keratinocytes. This suggests that there are corneal-specific mechanisms in DSB repair. This issue should be evaluated in future studies.

CRISPR/Cas9 itself has some probability of causing off-target mutations^22,23^. CRISPR RNA-guide endonucleases tolerate single and double mismatches in their sequences at the gRNA interface in bacterial cells^22^ and human cells^23^. Wu *et al*.^24^ reported that only 2 out of 12 samples had off-target mutations when they co-injected *Cas9* mRNA and a single gRNA into mouse zygotes with dominant mutations in *Crygc* that cause cataracts. Additionally, off-target mutations were detected at 1 of 10 potential OTS in the two samples. Thus, although off-target effects are an important safety issue for clinical use, they can be greatly reduced by a cautious gRNA sequence design^25,26^. Moreover, according to previous studies, gRNA does not cleave nonspecific targets with mismatches of 3 nt or more^23,27^. In our study, based on these findings, we made highly specific gRNAs using an off-target prediction tool. A T7 Endonuclease 1 cleavage assay was performed to examine off-targets effects, but the three predicted OTS were not detected in any sample (Fig. 3c).

In ocular tissues, several reports have demonstrated successful gene editing using the CRISPR/Cas9 system^28–31^. Wu *et al*. corrected a genetic disease in mice that show early-onset cataracts using non-homologous end joining and HDR^24^. The gene correction was conducted at the embryonic stage and cataracts occurred in 10 out of 12 mice. Wang *et al*.^32^ and Bakondi *et al*.^30^ successfully edited retinal genes by electroporation, and Hung *et al*.^28^ also successfully edited retinal genes using a virus delivery system. In the cornea, Courtney *et al*. reported the effectiveness of DNA cleavage by CRISPR/Cas9 for the treatment of cornea dystrophy caused by a KRT12 mutation^29^. To our knowledge, this study is the first to demonstrate *in vitro* gene correction in mutant human primary corneal cells using CRISPR/Cas9 and HDR. The cornea is an excellent tissue for the application of genome editing therapy owing to its accessibility and high amenability to naked plasmid DNA transfection via intrastromal injection^33^. Thus, gene editing is a radical GCD treatment and the *in vivo* application of this system is ideal for clinical settings in which conventional treatments are limited. In the future, it is necessary to develop safer and more efficient methods to modify local corneal genes *in vivo*.

In conclusion, we used CRISPR-Cas9-mediated HDR to correct the R124H mutation. Our data suggest that the approach is highly specific, with no observed off-target effects. Given the lack of effective treatment options for GCD2, this gene editing system is a potentially radical treatment for TGFBI-related corneal dystrophy and can be used to protect corneal opacities. The *in vivo* application of this system is an important future challenge.

## Methods

### Cell culture

Primary human corneal keratocytes of a GCD2 patient with a heterozygous TGFBI mutation (R124H) were isolated from a surgical specimen during deep anterior lamellar keratoplasty. Ethics approval for this work was obtained from the Institutional Review Board of the Inoue Eye Hospital and informed consent was obtained from the patient. All tissues were provided form Inoue Eye Hospital and no tissues were procured from prisoners. All the experimental methods were carried out in accordance with the guidelines verified and approved by the Ethics Committee of The University of Tokyo.

The cell culture method was described previously^34–36^. Briefly, the corneal epithelium was removed from the stroma of the surgical specimen by scraping with a razor blade. A stromal button was incubated overnight at 37 °C in basal medium, i.e. DMEM/F12 medium supplemented with B27 (Invitrogen, Carlsbad, CA, USA) containing 0.02% collagenase (Sigma-Aldrich, St. Louis, MO, USA). Subsequently, the digested tissue and cells were dispersed by pipetting and centrifuged at 800 × *g* for 5 min. After removing the supernatant, the keratocytes were resuspended in 1.0 mL of basal culture medium and seeded in culture dishes. The medium was changed every 2 days until the cells reached confluence. Second-passage cells were used in the subsequent transfection and other assays.

### gRNA design and CRISPR-Cas9 construct

Single gRNA targeting the R124H mutation site of the human *TGFBI* gene was designed using the CRIPSR design tool (publically available at http://crispr.mit.edu/, http://www.e-crisp.org/E-CRISP/). To construct the CRISPR-Cas9 plasmid targeting the human *TGFBI* gene, the complementary oligonucleotides hTGFBI gRNA-F and hTGFBI gRNA-R were phosphorylated using T4PNK (TAKARA, Kusatsu, Japan), annealed, and cloned into pSpCas9 BB-2A-GFP (PX458, plasmid #48138; Addgene, Cambridge, MA, USA) via the BbsI restriction sites. To utilize HDR to edit the human TGFBI R124H mutation, a 100-nt ssODN (hTGFBI R124H HDR ssODN) was designed to target the R124H mutation site.

The oligonucleotide sequences were as follows:

*hTGFBI* gRNA-F: 5′-CACCACTCAGCTGTACACGGACCACA-3′,

*hTGFBI* gRNA-R: 5′-AAACTGTGGTCCGTGTACAGCTGAGT-3′,

and *hTGFBI* R124H HDR ssDNA: 5′-GAGACCCTGGGAGTCGTTGGATCCACCACCACTCAGCTGTACACGGAC**CGTAC**GGAGAAGCTGAGGCCTGAGATGGAGGGGCCCGGCAGCTTCACCATCT-3′.

### Transfection and cloning

CRISPR-Cas9 constructs (2.5 µg per well) and ssODN (1 µg per well) were transfected into R124H primary cells using FuGENE (Promega, Madison, WI, USA) according to the manufacturer’s instructions and the cells were incubated for an additional 48 h. Images were obtained by fluorescence microscopy (BZ-9000; Keyence, Osaka, Japan). The cells expressing GFP were single-cell-sorted by FACS (Aria III, Becton-Dickinson, Franklin Lakes, NJ, USA) at 1 week after transfection. The sorted cells were then clonally expanded and analysed as described below.

### Indel analysis by restriction fragment length polymorphism (RFLP)

Total DNA was extracted from cells using the Nucleospin Kit (Takara Bio Inc.). Polymerase chain reaction (PCR) using specific primer sets (Forward: 5′-GTTGAGTTCACGTAGACAGGC-3′, Reverse: 5′-GACTCCCATTCATCATGCCCA-3′) was performed to amplify the DNA using the KOD FX Kit (KOD FX; Toyobo, Osaka, Japan) with the following temperature profile: 94 °C for 2 min, followed by 40 cycles of 98 °C for 10 s and 55 °C for 30 s, and 72 °C for 2 min. The PCR products were treated with the restriction enzyme BsiWI (New England Biolabs, Ipswich, MA, USA). One microgram of DNA was treated with 1 unit of enzyme and NE Buffer 2.1 at 55 °C for 15 min. The samples were analysed by electrophoresis on a 5% polyacrylamide TBE gel.

### DNA sequencing analysis

The target site (exon 4 of *TGFBI*) was amplified by PCR with primers (Forward: 5′-GTTGAGTTCACGTAGACAGGC-3′, Reverse: 5′-GACTCCCATTCATCATGCCCA-3′) targeting the genomic DNA of R124H-edited cells. After the purification of PCR products, the sequence of samples was analysed using a contract genome sequencing service. (Eurofins Genomics Inc., Tokyo, Japan).

### T7 Endonuclease I cleavage assay

The genome editing efficiency was investigated using a T7 endonuclease I cleavage assay. Genomic regions surrounding the target sites and potential off-target sites of gRNAs were amplified by PCR using Takara ExTaq (Takara Bio Inc.). Two hundred nanograms of gel-purified PCR products was re-suspended in NEB Buffer 2, and a hybridization reaction was performed using a thermocycler (BioRad, Hercules, CA, USA) with the following settings: 95 °C for 5 s, 95–85 °C at −2 °C/s, 85 °C for 30 s, 85–25 °C at −0.1 °C/s, 25 °C for 30 s, followed by maintenance at 4 °C. Five units of T7 endonuclease I (New England Biolabs) were added to digest the re-annealed DNA. After 2 h of incubation at 37 °C, DNA products were loaded on a 2% agarose gel and visualised after staining with ethidium bromide. Primers are listed in Table 1.

**Table 1:** Primer set used for the T7 endonuclease cleavage assay of potential off-target loci.

Column1	Forward	Reverse
#1	5′-ATGTCAGAAGTCCCGCTGTG-3′	5′-TGATGGGGTCAGAGGGCATA–3′
#2	5′-GCAGCAAAGCACTCAAGAGG-3′	5′-CAAACTTCTGCCTGGGCATC-3′
#3	5′-CTTCCTGCTCTGTGTTTAGCCA-3′	5′-ACCTCCAAGTTGAGCAGTGTC-3′
